# Formulation and stabilization of an *Arthrobacter* strain with good storage stability and 4-chlorophenol-degradation activity for bioremediation

**DOI:** 10.1007/s00253-017-8706-6

**Published:** 2018-01-18

**Authors:** Joakim Bjerketorp, Wilfred F. M. Röling, Xin-Mei Feng, Armando Hernández Garcia, Hermann J. Heipieper, Sebastian Håkansson

**Affiliations:** 1 Department of Molecular Sciences, Swedish University of Agricultural Sciences—SLU, Uppsala, Sweden; 2 Department Molecular Cell Physiology, VU University Amsterdam, Amsterdam, The Netherlands; 3 RISE Research Institutes of Sweden, Uppsala, Sweden; 4 Department Environmental Biotechnology, Helmholtz Centre for Environmental Research—UFZ, Leipzig, Germany

**Keywords:** Bioremediation, Bioaugmentation, *Arthrobacter*, Formulation, Microbial stabilization

## Abstract

Chlorophenols are widespread and of environmental concern due to their toxic and carcinogenic properties. Development of less costly and less technically challenging remediation methods are needed; therefore, we developed a formulation based on micronized vermiculite that, when air-dried, resulted in a granular product containing the 4-chlorophenol (4-CP)-degrading Gram-positive bacterium *Arthrobacter chlorophenolicus* A6. This formulation and stabilization method yielded survival rates of about 60% that remained stable in storage for at least 3 months at 4 °C. The 4-CP degradation by the formulated and desiccated *A. chlorophenolicus* A6 cells was compared to that of freshly grown cells in controlled-environment soil microcosms. The stabilized cells degraded 4-CP equally efficient as freshly grown cells in two different set-ups using both hygienized and non-treated soils. The desiccated microbial product was successfully employed in an outdoor pot trial showing its effectiveness under more realistic environmental conditions. No significant phytoremediation effects on 4-CP degradation were observed in the outdoor pot experiment. The 4-CP degradation kinetics from both the microcosms and the outdoor pot trial were used to generate a predictive model of 4-CP biodegradation potentially useful for larger-scale operations, enabling better bioremediation set-ups and saving of resources. This study also opens up the possibility of formulating and stabilizing also other *Arthrobacter* strains possessing different desirable pollutant-degrading capabilities.

## Introduction

Chlorophenols are widespread in the environment due to their use as paint and wood preservatives as well as general pesticides and disinfectants (herbicides, bactericides, insecticides, fungicides). These compounds are of environmental concern (Olaniran and Igbinosa 2011) and the US Environmental Protection Agency (EPA) lists several chlorophenols as priority pollutants due to their acute toxic and carcinogenic properties (ATSDR 2007). 4-chlorophenol (4-CP) is formed during chlorine treatment of waste water and chlorine bleaching of pulp in the paper industry as well as from more highly chlorinated phenols and degradation of phenoxy herbicides (Madsen and Aamand 1992; Czaplicka 2004). There are a number of physicochemical remediation methods to detoxify sites polluted with xenobiotics like 4-CP, but their widespread use is limited by being costly and technically challenging (Conesa et al. 2012).

Bioremediation is an alternative approach that makes use of the natural capabilities of living organisms to degrade and mineralize pollutants for nutrients and energy. Such methods have the potential to be sustainable and efficient as well as being cost effective. Typically, bioremediation processes employ microorganisms, plants (phytoremediation), or a combination of the two (rhizoremediation) (Dietz and Schnoor 2001; Kuiper et al. 2004).

Several approaches and technologies for microbial bioremediation have been tested and evaluated in microcosms, in greenhouse and in the field including biostimulation and bioaugmentation as previously reviewed (Megharaj et al. 2011; Cycon et al. 2017). Biostimulation or bioattenuation aims to promote activity of organisms naturally present in a certain environment whereas bioaugmentation instead relies on adding either a single species or a consortium of organisms, for example in order to accelerate the degradation of pollutants.

A previous study by Dams et al. (2007) on rhizoremediation using *Sphingobium chlorophenolicum* ATCC 39732—a well-known degrader of pentachlorophenol (PCP)—in combination with wheat plants demonstrated rapid degradation of PCP in soil (80% PCP degradation within 1 week) compared to either *S. chlorophenolicum* (80% PCP degradation within 2 weeks) or wheat (40% PCP degradation within 3 weeks) on their own. In situ bioremediation methods have been in use for more than 20 years but have so far yielded only limited success (reviewed in Pandey et al. 2009).

The practical implementation and usefulness of bioaugmentation of contaminated sites is constrained by the poor survival in situ of inoculated pollutant-degrading microbial isolates that otherwise have performed well in laboratory studies (Singer et al. 2005; Thompson et al. 2005). The development of a suitable formulation and stabilization methodology that will result in bioremediation products that meet a number of requirements such as efficacy, ease of use, low cost, and long shelf life is therefore essential. The current golden standard in microbial formulation and stabilization is to conserve the living cells in a state of arrested metabolism caused by a state of desiccation (anhydrobiosis) that can be successfully reversed through rehydration. Numerous microorganisms, invertebrates, and plants are capable of anhydrobiosis in nature for survival during periods of drought and desiccation. However, natural anhydrobionts are rare among known bacteria of interest for bioremediation applications.

Survival rates and storage qualities of formulated and stabilized microorganisms can be improved by providing an external carrier material that provides a protective environment throughout desiccation, storage, and rehydration (Vilchez and Manzanera 2011). The choice of drying technique, such as freeze-drying, spray-drying, or air-drying, can also affect survival rates and other qualities of the dry product as well as the overall costs. Freeze-drying is less suited for cost-sensitive large-scale applications of microorganisms due to relatively high energy inputs even though the desiccation survival rates may be very good (Melin et al. 2007). Harsher to the microorganisms but less expensive air-drying methods such as spray-drying, fluidized bed-drying, or even convectional air-drying therefore needs to be considered (Morgan et al. 2006; Santivarangkna et al. 2007).

With the applied limitation of low energy input and costs in mind, there are still many potential carrier materials and protectants to choose from when designing a microbial formulation (Santivarangkna et al. 2007). Vermiculite is an inorganic material with several desirable characteristics for formulation of microorganisms—it is inert, homogenous, well-defined, inexpensive, and known to be safe and commonly used in horticulture. Vermiculite is produced in larger quantities and at low costs (> 400,000 metric tons per year at a price from US$150 per metric ton according to the US Geological Survey 2016). Other researchers have also found vermiculite to be suitable as an inorganic carrier material for dry microbial formulations for use in biocontrol applications (Pesenti-Barili et al. 1991; Vidhyasekaran et al. 1997; Sarma et al. 2011). It has been reported that microbial cells physically adsorbs to the surface of vermiculite particles and can even aggregate small vermiculite particles into protective structures (Su et al. 2006; Müller and Défago 2006).

The genus *Arthrobacter* are Gram-positive, drought-tolerant, nutritionally versatile, aerobic bacteria that typically are found in soil, including the rhizosphere and the phyllosphere (Zhang et al. 2012; Santacruz-Calvo et al. 2013; Scheublin and Leveau 2013; Miranda-Ríos et al. 2015). The strain *Arthrobacter chlorophenolicus* A6, isolated from soil in CO, USA (Westerberg et al. 2000), can efficiently tolerate and degrade phenol and a variety of toxic substituted phenols such as 4-CP and 4-nitrophenol (4-NP) in high concentrations and over a wide temperature range (Westerberg et al. 2000; Backman and Jansson 2004; Unell et al. 2007). The species is classified as a class 1 microorganism by Ausschuss für Biologische Arbeitsstoffe/Committee on Biological Agents (ABAS), (TRBA466 2015).

In this study, our objectives were to investigate the feasibility of formulating and stabilizing a product based on desiccated *A. chlonophenolius* A6 in remediating soils contaminated with 4-CP and to assess the possible large-scale use of such an approach. Therefore, we investigated the 4-CP-degrading capacity of *A. chlorophenolicus* A6 cells either freshly grown or in a desiccated state, in both controlled-environment microcosms and outdoor pot trial. We also investigated the impact of plants (phytoremediation) on the degradation of 4-CP using rosemary (*Rosemarinus officinalis*) either alone or in combination with formulated and stabilized *A. chlorophenolicus* A6 cells. Rosemary is a sturdy, drought-tolerant small Mediterranean shrub with fine-threaded roots that was chosen as plant species to be applied in phytoremediation experiments within the EU-project BACSIN (Fernández et al. 2012, 2013).

## Materials and methods

### Bacterial strain and culture conditions

The *A. chlorophenolicus* A6 strain (DSM 12829) used in this study has been described previously (Westerberg et al. 2000). *A. chlorophenolicus* A6 was grown with shaking (140 rpm) at 25 °C in either lysogeny broth (LB) medium or in GM minimal medium (Alexander and Lustigman 1966) consisting of 2.1 g l^−1^ K_2_HPO_4_, 0.4 g l^−1^ KH_2_PO_4_, 0.5 g l^−1^ NH_4_NO_3_, 0.2 g l^−1^ MgSO_4_ heptahydrate, 23 mg l^−1^ CaCl_2_ dihydrate, and 2 mg l^−1^ FeCl_3_ hexahydrate with 0.13 g l^−1^ 4-chlorophenol (approximately 1 mM) as the sole source of carbon and energy. Agar (1.5% (*w*/*v*)) was added for growth on solid media. 4-CP containing agar plates were kept in the dark.

### Microbial formulation and stabilization by desiccation

*A. chlorophenolicus* A6 were grown in LB medium into stationary phase. Growth was monitored by optical density measurements at 600 nm. Stationary phase cells were collected by centrifugation at 4000×*g* for 20 min. The spent medium supernatant was decanted and filtered (0.2 μm). One part moist bacterial pellet by weight was mixed with 1.25 parts dry exfoliated vermiculite grade Micron, 0.1–1 mm (Dupré Minerals Ltd., Staffordshire, England) and 8.75 parts filtered spent medium.

The saturated bacteria/vermiculite mixture was then left to air-dry on aluminum trays for 48 h in a ventilated hood at relative humidity (RH) < 35% and ambient room temperature (approximately 22 °C).

Water content measurements were done using an 831 Karl Fisher coulometer (Metrohm Nordic, Bromma, Sweden). Water activity measurements were done with an AquaLab CX2 (Decagon Devices Inc., Pullman, WA, USA).

Viable count analysis was carried out with minimal media (GM) agar plates supplemented with 0.13 g l^−1^ 4-CP as the only source of carbon. To reconstitute the dry microbial product, the weight of water lost during desiccation was added to and allowed to disperse through the product at room temperature for at least 15 min. The reconstituted microbial product was further diluted 10 times (*w*/*v*) in physiological saline (0.85%) followed by rigorous full-speed vortexing for 60 s to detach the bacterial cells from the carrier. Two separated dilution series per sample were made with three platings per dilution. Colonies were counted after 6–7 days following inoculation. *A. chlorophenolicus* A6 colonies could easily be distinguished by their vigorous growth of 4-CP medium whereas any background colonies of bacteria somewhat tolerant to 4-CP were much smaller.

### Microcosm experiments

Two different soil types were used for the microcosm experiments. The first soil was collected from agricultural plots in Ter Munck near Leuven, Belgium (BACSIN reference soil; sand 14%, silt 71%, clay 15%;, 50°52′42″N, 4°39′24″E) (van Gestel et al. 2012). The second soil was a commercial unfertilized garden soil (Hasselfors Garden 0715) from Hasselfors Garden in Örebro, Sweden. Each soil (Ter Munck or Hasselfors) was inoculated as a batch with either freshly cultured or stabilized *A. chlorophenolicus* A6 to a final cell concentration of 2 × 10^6^ cfu g^−1^ dry soil in the Ter Munck soil and 2 × 10^8^ cfu g^−1^ dry soil in the Hasselfors soil. The soils were also supplemented with 4-CP to a final concentration of 130 μg g^−1^ dry soil, and the water content was adjusted to 60% of the water-holding capacity (WHC) of each specific soil. All additions were made in a rapid drop-wise manner with continuous mixing of the recipient soil. The soils were then mixed in plastic containers to ensure uniform distribution of bacteria, moisture, and pollutant. Soil aliquots corresponding to 5.0 g (dry weight) of either Ter Munck or Hasselfors soil was then transferred into 50-ml polypropylene tubes. The same soil (Ter Munck or Hasselfors) without *A. chlorophenolicus* A6 cells but mixed with the vermiculite carrier was used as a control for any background degradation of 4-CP. Tubes were incubated at 15 °C in the dark without agitation for the duration of the experiment. Duplicate samples were collected at time zero as well as after 4 h and 1, 2, 3, 4, 7, and 13 days for Ter Munck soil. For Hasselfors soil, triplicate samples were collected at time zero as well as after 1, 2, 3, 4, and 5 days. Tubes from each treatment were transferred to − 50 °C at each time point and stored frozen until later determination of 4-CP concentration. From each time point, one sample from the Ter Munk soil and triplicate samples from the Hasselfors soil were removed for the determination of viable cell density *A. chlorophenolicus* A6 expressed as colony-forming units (cfu). The soil samples were diluted 10 times (*w*/*v*) in physiological saline (0.85%) followed by the same procedure for determining cfu as described above.

### Outdoors pot trial

For the outdoor experiments, five different treatments were tested using different combinations of 4-CP, stabilized *A. chlorophenolicus* A6, and plants of rosemary (*Rosmarinus officinalis*). When used, 4-CP was added to the concentration of 130 μg ml^−1^. For each treatment, 3.1 kg (dry weight) of soil B (sieved through a 4-mm mesh) was put in 7-l pots and the water content of the soil was adjusted to 60% of the WHC. Each treatment regime involved different combinations of 4-CP, stabilized *A. chlorophenolicus* A6 (stabilized at a final cell concentration of 8 × 10^7^ cfu g^−1^ dry soil in Hasselfors soil), and live rosemary plants as described in Table 1. Each treatment was performed in triplicate.

**Table 1:** Different treatments tested during an outdoor 4-chlorophenol-degradation experiment

	Treatment:	1	2	3	4	5
4-chlorophenol (130 μg g^−1^ dry soil)		+	–	+	+	+
Stabilized *A. chlorophenolicus* A6 cells (7 × 10^7^ cfu g^−1^ dry soil)		+	+	−	+	−
Rosemary (*R. officinalis*)		+	+	+	−	−

Sampling was done by making two holes at the opposite sides of each pot using a small-diameter soil auger and then pooling these two subsamples into one single soil sample (approximately 10 g wet soil) per pot and sampling time. Samples were collected according to this procedure at the start of the experiment and then every day for 4 days and also at day 6. A portion of each sample was used for the determination of cfu, and the remainder of the sample was transferred to − 50 °C and stored frozen until further analysis. The soil samples were diluted 10 times (*w*/*v*) in physiological saline (0.85%) followed by the same procedure for determining cfu as described above.

### Extraction and HPLC analysis of 4-chlorophenol

Frozen soil samples were thawed and 5 g (dry weight) of soil from each sample was transferred to individual 50-ml polypropylene tubes by adding a total volume of 10 ml of 0.1 M NaOH dissolved in a 50% (*v*/*v*) methanol/water mixture followed by vigorous shaking at room temperature for 1 h in the dark.

Samples were then centrifuged at 1000×*g* for 15 min. One milliliter of supernatant from each sample was transferred to a 1.5-ml Eppendorf tube and acidified with 10 μl of concentrated (85% in H_2_O) phosphoric acid followed by centrifugation at 15,000×*g* for 10 min.

The concentration of 4-CP in each sample was then determined by HPLC analysis in Agilent 1100 system using a SB-18 column (Agilent ZORBAX) using a temperature gradient from 30 to 40 °C with a mobile phase consisting of 30 mM H_3_PO_4_ and methanol (2:3 *v*/*v*) and a flow rate of 1.2 ml^−1^ and the detection wavelength of 280 nm. Concentrations of the phenols and intermediates were calculated by comparison with external standards.

### Mathematical modeling

The 4-CP biodegradation in a Hasselfors Garden soil was mathematically modeled by non-linear regression using the GraphPad Prism 6.0 software in the non-linear regression section with 1000 iterations at maximum. An inverse sigmoidal model was used to describe the behavior of the 4-CP concentration in soil according to the Eq. (1)

1 $$ S=\frac{S_0}{\left(1+k{e}^{mt}\right)} $$

Where

S_0_: initial 4-CP concentration (*t* = 0)
*k*: dimensionless parameter (0 < *k* < 1)
*m*: time parameter or time constant (h^−1^)
*e*: Euler’s number

The viability was considered as a constant since the average value did not change significantly over time. The half-life time of 4-CP was determined as the time at which the initial substrate concentration had decreased to 50% ($\mathrm{S}=\frac{{\mathrm{S}}_0}{2}$). Therefore, using Eq. (1), we get

2 $$ \mathrm{S}=\frac{{\mathrm{S}}_0}{2}=\frac{{\mathrm{S}}_0}{\left(1+\mathrm{k}{\mathrm{e}}^{\mathrm{m}{\mathrm{t}}_{1/2}}\right)} $$

and from this mathematical relationship, we get the expression

3 $$ \left(1+\mathrm{k}{\mathrm{e}}^{\mathrm{m}{\mathrm{t}}_{1/2}}\right)=2 $$

which finally leads to

4 $$ {\mathrm{t}}_{1/2}=\frac{-\ln \left(\mathrm{k}\right)}{\mathrm{m}} $$

Through this equation, it is possible to calculate the half-life time of 4-CP in a soil augmented with *A. chlorophenolicus*.

## Results

### Survival, technical quality, and storage stability of stabilized *A. chlorophenolicus* A6

**Fig. 1:**
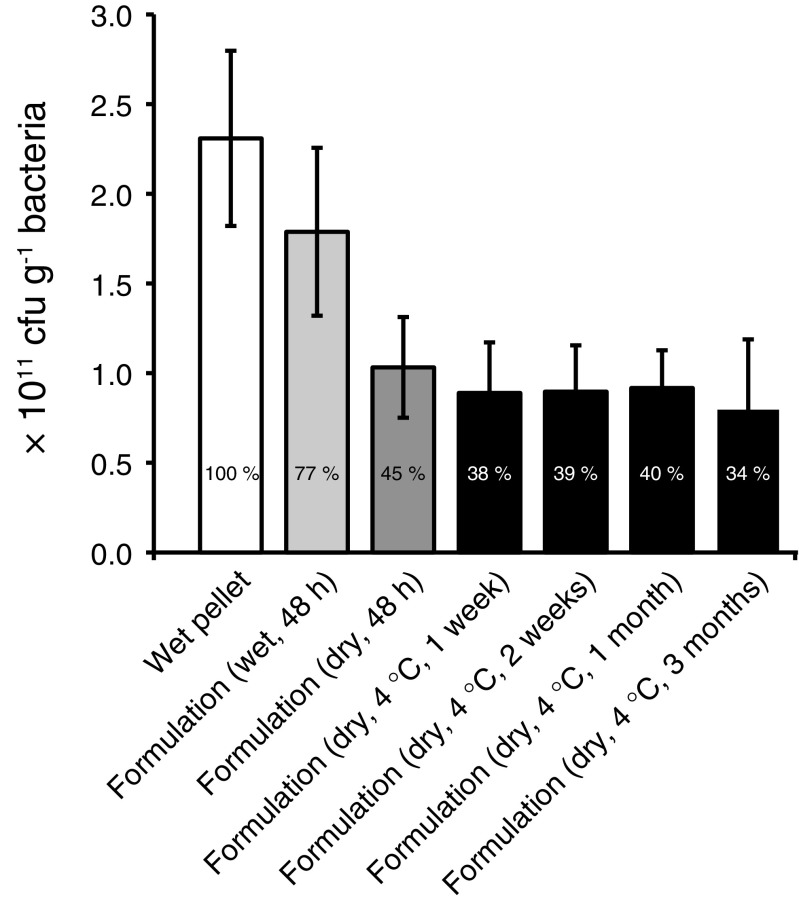
Viability of *A. chlorophenolicus* A6 stabilized with vermiculite at different lengths of storage at 4 °C. The arithmetic mean values with standard deviations of three separate drying experiments (batches) are shown. The percent survival as compared to fresh untreated cells are indicated

*A. chlorophenolicus* A6 cells were grown in rich LB medium to stationary phase, harvested and formulated by being mixed with a micron-grade vermiculite carrier. The average initial survival rate for triplicate experiments was found to be close to 60% after convectional air-drying for 48 h and subsequent rehydration as compared to the initial viability of the non-dried formulation. The dried product maintained a granular structure, and the granules were slightly larger than the original vermiculite granules. The average water content of the dried product was 4.2% as measured by Karl Fisher-titration. The water activity (a_w_) of the final stabilized product was 0.26, which corresponds to the water potential (Ψ) of − 184 MPa. Approximately 75% of the initial survival rate was preserved when stabilized *A. chlorophenolicus* A6 cells was stored at 4 °C for up to 3 months (Fig. 1).

### Degradation of 4-CP by *A. chlorophenolicus* A6 in controlled-environment microcosms

The dried microbial product was used for testing of its capability to degrade 4-CP in both controlled-environment microcosms with soil artificially contaminated (spiked) with 4-CP. In order to compare the capacity of the stabilized *A. chlorophenolicus* A6 cells to degrade 4-CP in soil with that of freshly cultured *A. chlorophenolicus* A6 cells, we used a simple microcosm experimental design with soil spiked with 130 μg of 4-CP g^−1^ incubated at 15 °C. The microcosms were inoculated with approximately equal numbers of stabilized bacteria or freshly grown bacteria. Soil lacking *A. chlorophenolicus* A6 cells but mixed with the vermiculite carrier was used as a control for background degradation of 4-CP.

The first soil to be tested originated from agricultural land in Ter Munck near Leuven in Belgium and is a well-characterized and commonly used reference soil (van Gestel et al. 2012). The Ter Munck soil had been hygienized by drying at 105 °C overnight prior to the experiment and was therefore assumed to be at least partially sterilized. The soil microcosm samples were spiked with 4-CP as described above and then inoculated with 2 × 10^6^ cfu g^−1^ dry soil of *A. chlorophenolicus* A6, stabilized or freshly grown.

**Fig. 2:**
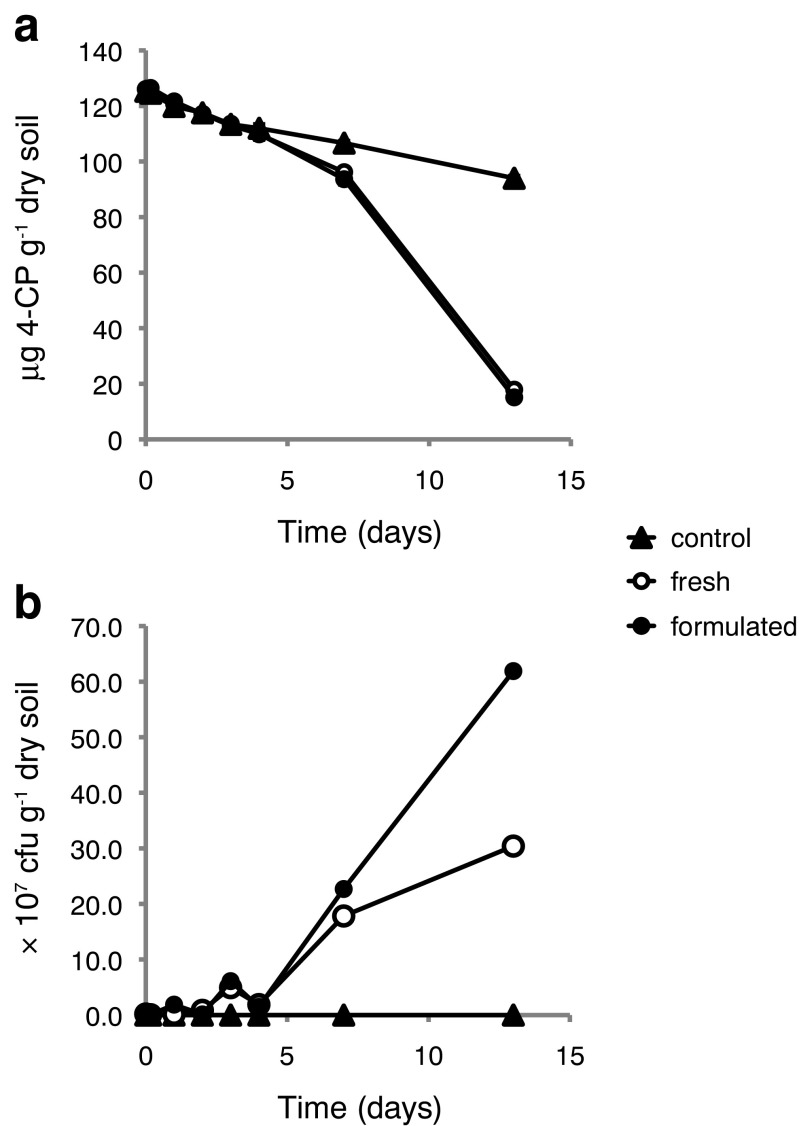
Microcosm experiment 1. Biodegradation of 4-CP in previously hygienized Ter Munck soil microcosms following inoculation with 2 × 10^6^ cfu of either freshly grown or stabilized *A. chlorophenolicus* A6 g^−1^ of dry soil. Vermiculite lacking *A. chlorophenolicus* A6 cells was added to the control. **a** Mean concentration of 4-CP in soil following inoculation. 4-CP concentration was measured in duplicate. Error bars indicate one standard deviation. **b** Number of viable *A. chlorophenolicus* A6 cells from a single sample per time point

After 13 days, the concentration of 4-CP was below 20 μg 4-CP g^−1^ dry soil both in the samples initially mixed with stabilized *A. chlorophenolicus* A6 cells and those mixed with fresh cells (Fig. 2a). The 4-CP concentration in the vermiculite-only control sample had decreased to 94.0 ± 0.8 μg 4-CP g^−1^ dry soil after 13 days, which corresponded to approximately 72% of the starting concentration. The number of *A. chlorophenolicus* A6 cells increased by more than two orders of magnitude during the experiment with a initial lag phase of at least 4 days (Fig. 2b). No colonies originating from bacteria endogenous to the Ter Munck soil were observed in the viable count assay for the non-inoculated vermiculite-only control (Fig. 2b).

A second set of soil microcosm experiments was carried out in order to compare the 4-CP degradation capacity of stabilized versus freshly grown *A. chlorophenolicus* A6 cells. This microcosm experiment used a commercially available, non-hygienized, and unfertilized garden soil from Hasselfors (Örebro, Sweden) that could be used in larger quantities for large-scale follow-up experiments without controlled laboratory conditions. The Hasselfors soil was spiked with 4-CP to a final concentration of 130 μg g^−1^ dry soil and was then inoculated with approximately equal numbers (2 × 10^8^ cfu g^−1^ of dry soil) of either stabilized or freshly grown *A. chlorophenolicus* A6 cells. A vermiculite-only soil lacking *A. chlorophenolicus* A6 cells was again used as a control for background degradation of 4-CP.

**Fig. 3:**
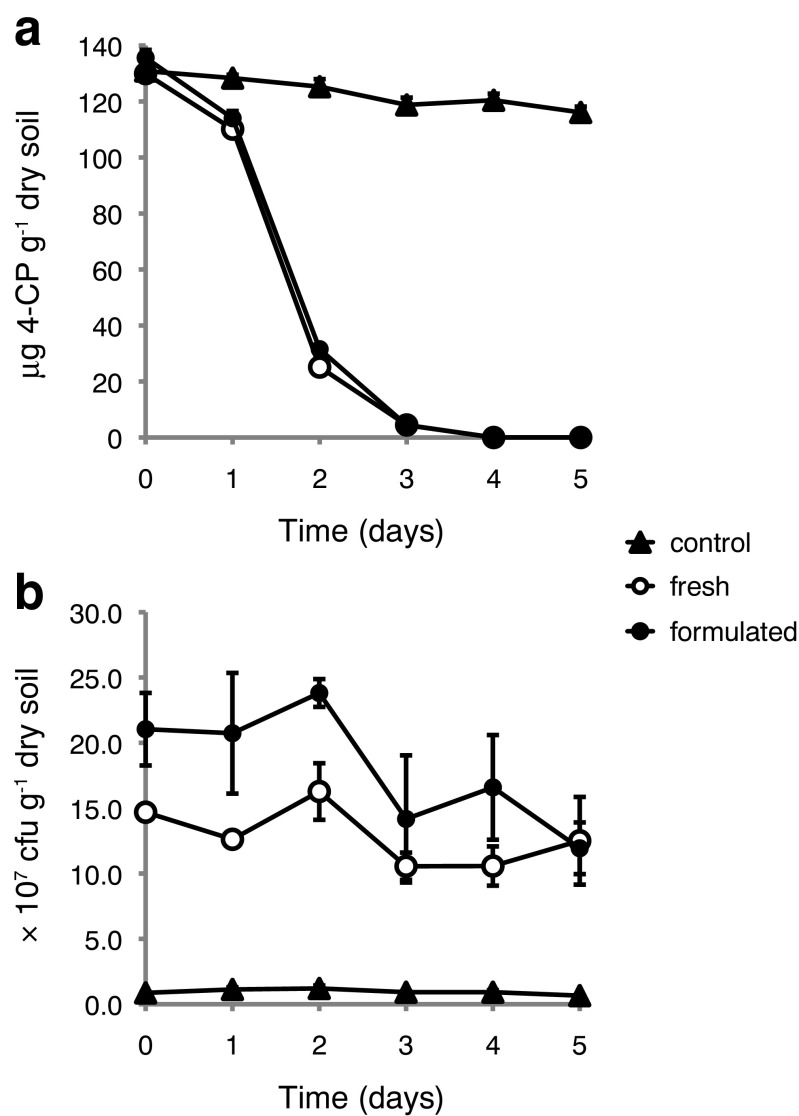
Microcosm experiment 2. Biodegradation of 4-CP in non-hygienized Hasselfors soil microcosms following inoculation with 2 × 10^8^ cfu of either freshly grown or stabilized *A. chlorophenolicus* A6 g^−1^ of dry soil. Vermiculite lacking *A. chlorophenolicus* A6 cells was added to the control. **a** Mean concentration of 4-CP in soil following inoculation. 4-CP concentration was measured in triplicate. Error bars indicate one standard deviation. **b** Mean number of viable *A. chlorophenolicus* A6 cells following inoculation. Viable cell counts were measured in triplicate. Error bars indicate one standard deviation

More than 95% of the initial amount of 4-CP was degraded already after 3 days in both the samples containing stabilized *A. chlorophenolicus* A6 cells and those containing fresh cells (Fig. 3a). After 4 days, 4-CP levels were below the limit of detection of the HPLC assay (2 μg ml^−1^ corresponding to 4 μg of 4-CP g^−1^ dry soil). The vermiculite-only control showed only a minor decrease (11%) in 4-CP concentration after 5 days. This agrees well with the vermiculite-only control in the Ter Munck soil microcosm experiment where 11% of 4-CP had been either degraded, absorbed, or evaporated after 4 days. Since the Ter Munck soil is hygienized, it is our opinion that the background 4-CP degradation activity probably is of an abiotic nature.

There was no obvious increase in *A. chlorophenolicus* A6 cell numbers in either the stabilized or the freshly grown cells (Fig. 3b). In fact, viable cell counts were negatively correlated with time (*r* = − 0.79 and − 0.54 for the stabilized and freshly grown *A. chlorophenolicus* A6 cells, respectively). However, viable cell count assays were only performed up to 5 days following inoculation and the Ter Munck soil had only demonstrated significant growth after 7 days.

Unlike the Ter Munck microcosm experiment, a background of viable cells corresponding to an average of (9.5 ± 2.0) × 10^6^ cfu g^−1^ dry soil was observed in the Hasselfors soil vermiculite-only control (Fig. 3b). These colonies were clearly distinguishable from *A. chlorophenolicus* A6 colonies by their much smaller size. These colonies were assumed to belong to bacteria that were either capable of limited 4-CP assimilation or utilized agar as a source of carbon. No attempt was made to characterize these colonies. There was no indication of increase in background cell numbers as measured by the viable count assay following 4-CP addition during the course of the experiment. Rather, it appeared to be a decline in cell numbers with time (*r* = − 0.54) possibly caused by 4-CP toxicity.

### Degradation of 4-CP by *A. chlorophenolicus* A6 during a pilot-scale outdoor pot experiment

In addition, we tested the ability of *A. chlorophenolicus* A6 to degrade 4-CP under more realistic conditions outside a controlled laboratory environment. We also wished to test whether a rhizoremediation approach with rosemary plants could accelerate or otherwise affect 4-CP degradation by *A. chlorophenolicus* A6. The experimental design consisted of five treatments with different combinations of 4-CP, *A. chlorophenolicus* A6 cells, and rosemary plants (Table 1). Only formulated *A. chlorophenolicus* A6 cells were used for the outdoor pot experiments since no difference in the capacity of fresh and stabilized cells to degrade 4-CP was observed in the microcosm experiments (Figs. 2a and 3a). The same initial concentration of 4-CP (130 μg g^−1^ dry soil) as in the microcosm experiments was used.

**Fig. 4:**
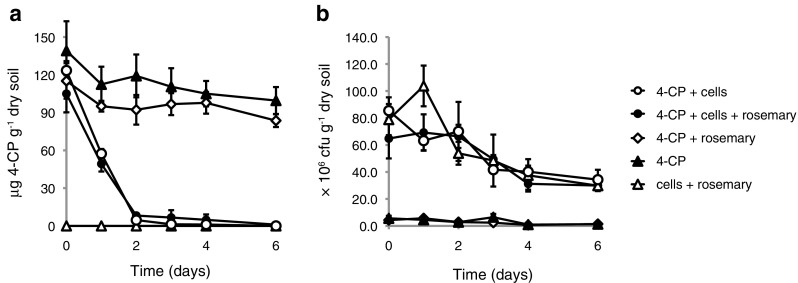
Outdoor pot trial. Biodegradation of 4-CP in non-hygienized Hasselfors soil outdoor pot experiments using different combinations of 4-CP (130 μg g^−1^ dry soil), stabilized *A. chlorophenolicus* A6 (8 × 10^7^ cfu g^−1^ dry soil) and rosemary plants. **a** Mean concentration of 4-CP in soil following initiation of the experiment. 4-CP concentration was measured in triplicate. Error bars indicate one standard deviation. **b** Mean number of viable *A. chlorophenolicus* A6 cells following initiation of the experiment. Viable cell counts were measured in triplicate. Error bars indicate one standard deviation

Degradation of 4-CP was observed already after 1 day in the two treatments that included *A. chlorophenolicus* A6 and 4-CP (Fig. 4a). 4-CP levels decreased below 10% of the starting concentration after 2 days and below the limit of detection after 6 days. The roots of the rosemary plants grew to the full depth and width of the pots during the time of the experiment.

It can be concluded that the presence of the plants did not affect the number of 4-CP degrading microorganisms in this experimental set-up (Fig. 4a). In fact, net growth of *A. chlorophenolicus* A6 was not observed in any of the combinations used in the outdoor trial (Fig. 4b). However, the degradation of 4-CP was faster than in the 15 °C Hasselfors soil microcosms (compare Figs. 3a and 4a).

There was a minor endogenous bacterial flora of small colonies observed using the viable count assay for the two treatments that excluded *A. chlorophenolicus* A6 cells. In the treatment with 4-CP and rosemary plants, an average cell count of (3.9 ± 1.8) × 10^6^ cfu g^−1^ dry soil was observed during the course of the experiment. In the treatment with 4-CP only the average cell count was (3.6 ± 2.3) × 10^6^ cfu g^−1^ dry soil. As in the Hasselfors soil microcosm experiment, there was a negative trend in cell numbers of the background microbial flora during the 6-day experiment (*r* = − 0.82 and − 0.67 for treatments [4-CP with rosemary] and [4-CP], respectively). This observation agrees well with what was observed for the non-inoculated Hasselfors microcosm experiment (Fig. 2b).

### Modeling 4-CP biodegradation by *A. chlorophenolicus* A6

Mathematical modeling of biodegradation in general has been extensively applied using both differential and integral approaches (Schmidt et al. 1985; Sahoo et al. 2011). Depending on the complexity of the biological system studied, modeling by using classical differential equations systems could introduce significant errors, even more so when there are only a few experimental data points. The use of an integral or non-linear regression approach would be an alternative in such a case. This implies the construction of a model with the minimal number of parameters required to explain, as simple as possible, the behavior of the system under study. Since in our kinetics experiments only a few measurements were made per experiment (*n* < 10), we decided to describe the biodegradation and growth data using a simplistic model. Due to the biodegradation pattern observed herein, we used an inverse sigmoidal model that describes the experimental data of 4-CP degradation well (Table 2). The half-life of 4-CP is a point estimator of the biodegradation kinetics, meaning that the biodegradation process will be faster as 4-CP half-life decreases. According to this model, the 4-CP biodegradation is faster as *k* increases and *m* decreases according to Eq. (4). Table 2 shows that 4-CP biodegradation is more rapid in the outdoor pots trials than in the microcosms. Since the viability does not change over time, it is likely that cells are only using 4-CP as a substrate for maintenance.

**Table 2:** Parameters estimated from the biodegradation kinetic model for 4-CP

Treatment	*k*	*m* (h^−1^)	t_1/2_ (h)	R^2^
Microcosm Hasselfors Garden in tubes (vermiculite inoculum)	(1.1 ± 0.1) × 10^−2^	(1.20 ± 0.03) × 10^−1^	38.0 ± 1.0	0.9991
Microcosm Hasselfors Garden in tubes (liquid inoculum)	(8 ± 1) × 10^−3^	(1.30 ± 0.04) × 10^−1^	37.1 ± 1.1	0.9988
Microcosm Hasselfors Garden in pots (4-CP + cells + Rosemary)	(4.4 ± 1) × 10^−2^	(1.3 ± 0.3) × 10^−1^	23.5 ± 6.1	0.9635
Microcosm Hasselfors Garden in pots (4-CP + cells)	(1.9 ± 1) × 10^−2^	(1.7 ± 0.3) × 10^−1^	23.2 ± 4.3	0.9946

## Discussion

The present study was designed to develop a low-cost workable formulation and stabilization of *A. chlorophenolicus* A6 for the degradation of 4-CP in contaminated soils. To this end, we developed a simple growth protocol not dependent on the target pollutant, followed by a formulation protocol based on micronized vermiculite. The formulation and stabilization procedure did not require any specialized equipment beyond the capacity to air-dry the formulation at < 35% RH and ambient room temperature. The dry microbial product showed a high stability and high survival rates of the bacteria also after 3 months of storage at 4 °C.

The kinetics of 4-CP degradation were essentially identical between stabilized and freshly grown *A. chlorophenolicus* A6 cells irrespective of the different soils used in the microcosm experiments (Figs. 2a and 3a). The Ter Munck soil had been hygienized by oven-drying prior to the experiment while the Hasselfors soil had not. The inoculum size of the Hasselfors soil microcosm experiment was a hundred-fold larger than the one used in the Ter Munck soil microcosm experiment. The higher cell input to the Hasselfors soil was done in order to find out if we could increase the degradation rate of CP-4 and also to guide us in planning of the outdoors pot trial. The numbers of bacteria used throughout our study is well within the range reported for inoculum sizes suitable for efficient degradation of pesticides in soil (Cycon et al. 2017).

The growth of *A. chlorophenolicus* A6 observed in the Ter Munck soil (Fig. 2b) could be a direct result of the drying process of the soil at 105 °C over night prior to the inoculation. This hygienization of the soil would have allowed the inoculum of *A. chlorophenolicus* A6 to colonize the soil without any significant competition from endogenous microorganisms. It is also possible that hygienization of the soil would delay the degradation of 4-CP since the *A. chlorophenolicus* A6 cells would prioritize more accessible carbon sources that no longer are subject to competition from the endogenous microbiome.

When *A. chlorophenolicus* A6 was inoculated into untreated Hasselfors soil (Fig. 3a), no increase in viable cell counts was observed, which would support this argument. However, the increase in *A. chlorophenolicus* A6 cell numbers in the Ter Munck soil occurred only after the 4-day time point, and therefore, the lack of detectable growth of *A. chlorophenolicus* A6 in Hasselfors soil may simply be due to termination of the experiment already after 5 days due to the fast 4-CP degradation in this experiment. The rapid degradation of 4-CP in non-hygienized Hasselfors soil under both controlled microcosm conditions (Fig. 2a) and outdoor pot experiments (Fig. 4a) support such a hypothesis. In addition, the weather during the outdoor pot experimental period was unusually hot with a mean value of 21.5 °C (high 34.5 °C, low 11 °C). Naturally, this could also have impacted the degradation kinetics.

Under natural non-sterile conditions, the endogenous microbiome of the soil is expected to be highly competitive for readily available carbon sources, so it would be expected that *A. chlorophenolicus* A6 would primarily utilize 4-CP as a carbon source with little outside competition. The initial level of 4-CP (approximately corresponding to 1 mM) spiked into the Hasselfors soil may be too low to support any significant growth of *A. chlorophenolicus* A6, which is supported by the viable cell measurements in both the microcosm experiment (Fig. 3b) and the outdoor pot experiment (Fig. 4b).

Also, others have investigated the degrading capacity of an *Arthrobacter* strain capable of degrading simazine by adding various carbon sources as well as the pollutant to microcosms (Guo et al. 2014). They found that biostimulation in combination with bioaugmentation could enhance the biodegradation rate of this herbicide using their *Arthrobacter* strain. We have yet to try if biostimulation in some way could increase the degradation rate of 4-CP and/or the growth of *A. chlorophenolicus* A6. However, there is also the risk that other available carbon sources will be the preferred choice and then the degradation rate of the target pollutant may decrease until the depletion of the more preferred substrate (Unell et al. 2008).

We evaluated the impact of potential phytoremediation in our study. However, no significant effect by the rosemary plants on 4-CP degradation either alone or in combination with *A. chlorophenolicus* A6 was observed in the outdoor pot experiment (Fig. 4a) even though the log octanol-water partition coefficient for 4-CP (2.39) is within the range that would make 4-CP bioavailable to plants (Dietz and Schnoor 2001).

In order to use the empirical data obtained in this study for future up-scaling of the bioremediation process, we modeled the 4-CP degradation pattern. We have used a non-linear regression approach since the 4-CP biodegradation follows an inverse sigmoidal pattern with two constants or empirical parameters that allow the determination of the half-life of the pollutant.

The main estimator of 4-CP biodegradation kinetics was the half-life calculated through Eq. (4). The half-life for a pollutant is conceptually defined as the time required for a substance to decrease to half of its initial concentration (Agarry et al. 2013; Agarry and Oghenejoboh 2015). The biodegradation of 4-CP was faster in the pots trials (lower t_1/2_) compared to the microcosm trials (Table 2) likely due to uncontrolled environmental parameters such as soil relative moisture and temperature that fluctuated during the experiment, thus impacting on the observed biodegradation kinetics.

The model can be used to predict the 4-CP biodegradation at larger scale (e.g. field treatments in the same type of soil). Thus, starting from a similar relative inoculum size of *A. chlorophenolicus* A6, the same biodegradation pattern would be expected and therefore the time to degrade all the pollutant could be calculated ahead using the model, which leads to the possibility of better bioremediation set-ups and saving of resources.

In addition, since both parameters *m* and *k* are dependent on the temperature, it would be interesting to determine the behavior of the biodegradation profile at different constant temperatures. That is, knowing the kinetic parameters of the system at different temperatures would allow for a more reliable prediction of the 4-CP biodegradation by *A. chlorophenolicus*.

The successful formulation and stabilization of *A. chlorophenolicus* A6 and the subsequent bioaugmentation of 4-CP degradation using such a microbial product open up the possibility of similar approaches for other *Arthrobacter* strains possessing various desirable pollutant-degrading capabilities. The use of genetically modified microorganisms in order to combine abilities for pollutant mineralization with abilities to produce compatible solutes for increased survival rates during formulation and stabilization is an interesting possibility for future work.
